# Discovery of CMNPD31124 as a novel marine-derived PKMYT1 inhibitor for pancreatic ductal adenocarcinoma therapy: computational and biological insights

**DOI:** 10.3389/fphar.2025.1569765

**Published:** 2025-04-09

**Authors:** Chaojie Huang, Ting Wang, Rui Chen, Yunyun Xu

**Affiliations:** ^1^ Department of Colorectal Surgery, Sir Run Run Shaw Hospital, Zhejiang University School of Medicinesla, Hangzhou, China; ^2^ The Third Affiliated Hospital, Guangzhou University of Chinese Medicine, Guangzhou, China; ^3^ College of Life Sciences and Health Engineering, Jiangnan University, Wuxi, China; ^4^ General Surgery, Cancer Center, Department of Gastrointestinal and Pancreatic Surgery, Zhejiang Provincial People’s Hospital (Affiliated People’s Hospital), Hangzhou Medical College, Hangzhou, China

**Keywords:** PDAC, marine natural products, covalent inhibitor, molecular docking and dynamics, biological evaluations, toxicity assessment

## Abstract

Pancreatic ductal adenocarcinoma (PDAC) remains one of the deadliest cancers due to its late diagnosis, resistance to therapy, and a dismal 5-year survival rate of only 12%. Overexpression of PKMYT1—a key regulator of the cell cycle—correlates with poor patient outcomes, making it a promising therapeutic target. In this study, we identify CMNPD31124, a novel marine-derived indole alkaloid, as a potent PKMYT1 inhibitor. Molecular docking revealed that CMNPD31124 has superior binding affinity compared to the reference compound Cpd 4, forming robust interactions with critical residues such as CYS-190, TYR-121, and GLY-122. Molecular dynamics simulations further demonstrated its stable binding conformation and dynamic adaptability, with Chai-1 modeling supporting a covalent binding mechanism at the PKMYT1 active site. Importantly, *in vitro* assays showed that CMNPD31124 exhibits an IC_50_ of 18.6 μM in MiaPaCa-2 cells and 31.7 μM in BXPC3 cells, while concentrations up to 80 μM did not significantly affect normal pancreatic cells. Despite these promising results, toxicity predictions indicate potential hepatotoxicity and neurotoxicity, highlighting the need for further structural optimization. This work lays a solid foundation for the rational design of PKMYT1 inhibitors by integrating computational methods with insights from marine natural products.

## 1 Introduction

Pancreatic cancer (PDAC) is a highly lethal malignancy with a poor prognosis. In 2024, it is estimated that there will be approximately 66,440 new cases and 51,750 deaths in the United States. Globally, pancreatic cancer accounted for 496,000 new cases and 466,000 deaths in 2020, ranking among the top causes of cancer-related mortality (Siegel et al., 2024). The incidence and mortality rates of PDAC have been steadily rising, driven by factors such as an aging population, smoking, obesity, and genetic predispositions (Cai et al., 2021). Despite advancements in medical research, the 5-year relative survival rate remains approximately 12%, primarily due to late-stage diagnoses and the aggressive nature of the disease (Zeng et al., 2024). PDAC’s unique tumor microenvironment (TME), characterized by immunosuppressive stromal cells, tumor-associated macrophages (TAMs), and myeloid-derived suppressor cells (MDSCs), further complicates therapeutic interventions and contributes to resistance against conventional treatments (Herting et al., 2021). These challenges underscore the urgent need for novel therapeutic approaches that address both genetic heterogeneity and the TME’s complexity.

Protein kinase membrane-associated tyrosine/threonine 1 (PKMYT1) has emerged as a promising therapeutic target in PDAC due to its pivotal role in regulating the cell cycle (Yang et al., 2024). As a member of the WEE kinase family, PKMYT1 uniquely phosphorylates CDK1 at both THR14 and TYR15, serving as a critical checkpoint for mitotic entry, particularly under replication stress (Asquith et al., 2020). Unlike its nuclear counterpart WEE1, PKMYT1 is primarily cytoplasmic, associated with the Golgi apparatus and endoplasmic reticulum (Tomović Pavlović et al., 2024). In PDAC, PKMYT1 is significantly overexpressed and strongly correlates with poor prognosis. Its inhibition induces mitotic catastrophe by disrupting CDK1 activity, selectively targeting cancer cells that rely heavily on the G2/M checkpoint while sparing normal cells, making it an attractive therapeutic target (Wang et al., 2024a).

Recent advancements in PKMYT1 inhibitor development have further reinforced its therapeutic potential (Yang et al., 2024). RP-6306, a first-in-class orally bioavailable PKMYT1 inhibitor, has demonstrated significant antitumor efficacy in preclinical and clinical studies (Szychowski et al., 2022). PKMYT1 inhibition is particularly effective in PDAC, where it plays a crucial role in regulating the G2/M checkpoint during mitosis, making it a promising target for therapeutic intervention. The development of PKMYT1 inhibitors represents a significant advancement in addressing the challenges of PDAC treatment and highlights the potential to selectively target tumor cells while sparing normal tissues, thus enhancing therapeutic efficacy in PDAC patients (Tomović Pavlović et al., 2024).

Marine natural products represent a unique and rich resource for drug development, offering unparalleled structural diversity and bioactivity compared to synthetic and terrestrial-derived compounds (Banday et al., 2024). The extreme marine environment drives the evolution of structurally complex molecules with novel mechanisms of action, making them particularly appealing for targeting challenging proteins like PKMYT1. For example, the anticancer drug trabectedin (Yondelis), originally isolated from the sea squirt Ecteinascidia turbinata, demonstrates potent activity against soft tissue sarcomas and ovarian cancer (Monk et al., 2012). Similarly, eribulin mesylate (Halaven), a synthetic derivative of halichondrin B from the marine sponge Halichondria okadai, is approved for the treatment of metastatic breast cancer (McBride and Butler, 2012). In the field of antimicrobials, the antibiotic daptomycin (Cubicin), derived from the marine bacterium *Streptomyces* roseosporus, has proven highly effective against multidrug-resistant Gram-positive infections (Steenbergen et al., 2005). These examples underscore the potential of marine natural products to address limitations in traditional drug discovery pipelines, particularly by providing scaffolds with novel mechanisms of action and superior pharmacological properties.

The advent of computer-aided drug design (CADD) has revolutionized the drug discovery process, enabling the rapid identification and optimization of lead compounds (Niazi and Mariam, 2024). Techniques such as molecular docking, molecular dynamics simulations, and binding free energy calculations facilitate the prediction of ligand-protein interactions with high accuracy, reducing the reliance on costly and time-consuming experimental screening (Rampogu et al., 2022; Xu et al., 2024a). In addition, machine learning-based approaches, such as the integration of K nearest neighbors with nonnegative matrix factorization (KNN-NMF), have demonstrated potential in predicting biomolecular associations, including circRNA-disease relationships, which further highlights the applicability of computational techniques in biomedical research (Wang et al., 2023). Similarly, structural perturbation-based matrix completion methods (SPCMLMI) have been successfully applied to infer lncRNA-miRNA interactions, demonstrating the potential of network-based predictive modeling for uncovering complex biomolecular relationships (Wang et al., 2022b). By integrating structural biology and computational modeling, CADD provides a powerful platform to accelerate the development of selective and effective inhibitors for therapeutic targets such as PKMYT1. In parallel, biological evaluations of CMNPD31124 demonstrated promising antitumor activity in PDAC cell lines, while showing low toxicity to normal pancreatic cells, supporting its potential as a therapeutic candidate.

In this study, we combined the advantages of marine natural products and CADD approaches to identify and evaluate CMNPD31124, a novel PKMYT1 inhibitor with promising therapeutic potential against PDAC. The results demonstrate the effectiveness of integrating computational and structural insights to guide the rational design of innovative anticancer agents.

## 2 Method

### 2.1 Preparation of PKMYT1 structure and redocking of Cpd 4

Obtained from the Protein Data Bank, the crystal structure of PKMYT1 with the reference inhibitor Cpd 4 (PDB ID 8WJY) (Wang et al., 2024b) displays a resolution of 1.88 Å. Through the Schrodinger 2024-1 suite’s protein preparation wizard (Schrödinger Release 2024-3: Protein Preparation, Schrödinger, LLC, New York, NY, 2024) (Sastry et al., 2013) and prime module, hydrogen atoms are added, missing loops filled, termini capped, charge states adjusted, and inappropriate H-bond orders fixed. Various steric strains and heavy atoms up to 0.3 Å were eliminated using the restrained energy minimization OPLS 2005 force field (Jorgensen et al., 1996).

### 2.2 Compound library preparation

Accessed from the freely available CMNPD, this open-access, manually curated database is specifically designed for research on marine natural products. It offers comprehensive details on chemical compounds, including their physicochemical and pharmacokinetic characteristics, standardized data on biological activities, systematic taxonomy, geographic distribution of the source organisms, and extensive references to relevant literature (Lyu et al., 2021). Using the LigPrep tool (Schrödinger Release 2024-3: LigPrep, Schrödinger, LLC, New York, NY, 2024) (Greenwood et al., 2010), the ligand molecules were prepared, followed by geometric minimization employing the OPLS 2005 force field, ensuring specified chirality is retained at a pH range of 7.0 ± 2.0.

### 2.3 Structural similarity calculations between CMNPD compounds and Cpd 4

Cpd 4 served as the reference molecule, and the Shape Screening tool (Schrödinger Release 2024-3: Phase, Schrödinger, LLC, New York, NY, 2024) from Schrödinger was utilized for the screening process (Sastry et al., 2011). Two primary similarity measures were employed: shape similarity and chemical feature similarity. Shape similarity was evaluated using the ROCS algorithm (Kearnes and Pande, 2016), which quantified molecular volume overlap to determine structural alignment. Chemical feature similarity focused on functional groups, including hydrogen bond donors, acceptors, and hydrophobic regions, through chemical feature mapping methods.

### 2.4 Receptor grid generation and molecular docking

The Receptor Grid Generation tool (Schrödinger Release 2024-3: Glide, Schrödinger, LLC, New York, NY, 2024) was employed to define the grid at the Cpd 4 binding site on the target protein (Mohamed et al., 2022). The receptor grid delineates the interaction zone between the ligand and the protein. This process involved selecting the co-crystallized ligand within the binding site of PKMYT1 to accurately identify the interaction region. Molecular docking was performed using Glide docking module, utilizing both standard precision (Cross et al., 2009) and Prime MM-GBSA (Bashford and Case, 2000). The prepared compounds were docked to the protein to identify those with the lowest docking scores. During docking, the compounds were treated as flexible, while the protein structure was kept rigid.

### 2.5 All-atom molecular dynamics simulations

In the initial stage, all-atom MD simulations were performed using the Desmond module (Schrödinger Release 2024-3: Desmond, Schrödinger, LLC, New York, NY, 2024) (Bowers et al., 2006). The docked complexes were placed in a cubic water box with a 10 Å buffer, utilizing the simple point charge (SPC) water model and 0.15 M sodium chloride (NaCl) to simulate physiological conditions. Electrostatic interactions were calculated using the particle-mesh Ewald (PME) method, while van der Waals (vdW) interactions employed a 9.0 Å cutoff. Following solvation, the system underwent minimization and equilibration according to Desmond’s standard protocols in both constant number of particles, volume, and temperature (NVT) and constant number of particles, pressure, and temperature (NPT) ensembles.

Subsequently, a 100 nanoseconds (ns) NPT simulation was conducted under periodic boundary conditions using the OPLS 2005 force field. Temperature and pressure were maintained at 300 K (K) and 1 atm (atm) using the Nosè-Hoover chain thermostat and the Martyna-Tobias-Klein barostat. This initial phase incorporated a multi-step approach, beginning with Brownian dynamics at 10 K, progressing through a series of restrained and unrestrained NVT and NPT simulations, and concluding with a 1,000 ns NPT simulation under the same temperature and pressure parameters.

### 2.6 Prediction of protein-ligand complex structures using Chai-1 modeling framework

The Chai-1 modeling framework (https://github.com/chaidiscovery/chai-lab) was utilized to predict the complex structure between the protein and the ligand. The process began with the preparation of the protein sequence in FASTA format and the ligand’s SMILES representation. These inputs were provided to Chai-1, which integrates protein structure prediction and ligand-binding modeling. The modeling workflow generated five predicted structures of the protein-ligand complex, ranked based on their structural feasibility and interaction characteristics.

During the modeling process, Chai-1 incorporated advanced algorithms to simulate the folding of the protein and its interaction with the ligand, ensuring a comprehensive prediction of the binding conformation. The resulting models were further analyzed to identify critical binding interactions, including hydrogen bonds and hydrophobic contacts, as well as potential covalent interaction sites. This approach provided detailed insights into the binding mechanism and facilitated the evaluation of the ligand’s potential as a targeted inhibitor.

### 2.7 Culture conditions for MiaPaCa-2, BXPC3, and hTERT-HPNE cell lines

MiaPaCa-2 cells (human pancreatic carcinoma, ATCC^®^ CRL-1420™) are cultured in high-glucose Dulbecco’s Modified Eagle Medium (DMEM) supplemented with 10% fetal bovine serum (FBS) under conditions of 37°C and 5% CO2. BXPC3 cells *(human pancreatic adenocarcinoma, ATCC^®^ CRL-1687™)* are maintained in Roswell Park Memorial Institute (RPMI) 1,640 medium supplemented with 10% FBS, also under conditions of 37°C and 5% CO2. hTERT-HPNE cells *(human telomerase-immortalized pancreatic ductal epithelial cells, ATCC^®^ CRL-4023™)* are grown in Keratinocyte Serum-Free Medium (K-SFM) supplemented with 50 μg/mL bovine insulin and 5 ng/mL epidermal growth factor (EGF) at 37°C and 5% CO2.2.8 CCK-8 Assay in MiaPaCa-2, BXPC3, and hTERT-HPNE Cell Lines.

The CCK-8 assay was employed to assess cell viability, evaluating the proliferative or inhibitory effects of CMNPD31124 on the cells. Once the cells reached approximately 80% confluence, CMNPD31124 was added at various concentrations ranging from 0.1 μM to 100 μM. The cells were incubated with the drug for 48 h. After the treatment period, the CCK-8 reagent was added according to the manufacturer’s instructions, and the plates were gently mixed. The plates were then incubated at 37°C with 5% CO_2_ for an additional 2 h, depending on cell activity and drug concentration. Absorbance was measured at 450 nm using a microplate reader. Control wells without drug treatment and blank wells containing only media were included to measure baseline absorbance. Cell viability was calculated by comparing the absorbance values of treated cells with the untreated control group. The IC_50_ values were derived from dose-response curves to determine the inhibitory effects of CMNPD31124 on cell viability.

### 2.8 Toxicity predictions

The ProTox-II online server (Banerjee et al., 2018) was used to determine the acute toxicity class, LD_50_, hepatotoxicity, carcinogenicity, mutagenicity, cytotoxicity, and immunotoxicity of the compounds.

## 3 Results

### 3.1 Compound similarity screening and physicochemical property evaluation based on the structure of Cpd 4

**FIGURE 1:**
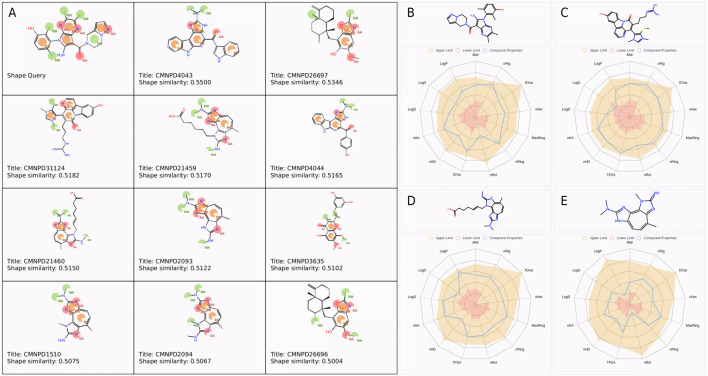
Pharmacophore features and physicochemical property analysis of selected compounds. **(A)** Pharmacophore features and similarity scores of selected compounds. Base feature spheres represent hydrogen bond acceptors (pink), hydrogen bond donors (blue), hydrophobic regions (green), and aromatic rings (orange). **(B–E)** Physicochemical property radar charts for **(B)** Cpd 4, **(C)** CMNPD31124, **(D)** CMNPD21459, and **(E)** CMNPD1510.

A total of 11 compounds with a similarity score greater than 0.5 were identified from the CMNPD database after shape-based screening, as shown in Figure 1A. Overall, CMNPD4043 exhibited the highest similarity to Cpd 4, whereas CMNPD26696 showed the lowest similarity among the candidates. These similarities could provide valuable insights for subsequent structural optimization or activity prediction.

To assess the drug-likeness potential of these compounds, their physicochemical properties were evaluated. The screening criteria included a molecular weight range of 100–600 Da, LogP between 0 and 3, LogS from −4 to 0.5, and TPSA below 140 Å^2^, among other properties. The compounds that met the theoretical threshold ranges are presented in Figures 1B–E. The molecular weights of Cpd 4 (431.21), CMNPD31124 (476.19), CMNPD21460 (382.21), and CMNPD1510 (256.14) were all within the theoretical range of 100–600. Regarding the number of hydrogen bond acceptors (nHA), Cpd 4 and CMNPD31124 showed the highest value of 9, followed by CMNPD21460 with 8, and CMNPD1510 with the lowest value of 6, all within the theoretical range of 0–12. For hydrogen bond donors (nHD), CMNPD31124 had the highest value of 5, followed by Cpd 4 with 3, while CMNPD21460 and CMNPD1510 had 2 each, all fitting the theoretical range of 0–7.

In terms of the number of rotatable bonds (nRot), CMNPD31124 and CMNPD21460 exhibited relatively higher values of 6 and 7, respectively, while Cpd 4 and CMNPD1510 had values of 3 and 1, respectively. The number of rings (nRing) was 5 for both Cpd 4 and CMNPD31124, and 3 for CMNPD21460 and CMNPD1510, consistent with the theoretical range of 0–6. The maximum ring size (MaxRing) was 5 for Cpd 4 and CMNPD31124, and 3 for CMNPD21460 and CMNPD1510.

The number of heteroatoms (nHet) ranged within the theoretical limits of 0–8, with Cpd 4, CMNPD31124, and CMNPD21460 having 5 each, and CMNPD1510 having 4. All compounds had a formal charge (fChar) of 0, indicating no net charge and meeting the theoretical range of −1 to 1. The number of rigid bonds (nRig) was 9 for Cpd 4, 8 for CMNPD31124 and CMNPD21460, and 6 for CMNPD1510, all within the range of 0–10.

The topological polar surface area (TPSA) values were 75.69 for Cpd 4, 97.99 for CMNPD31124, 74.62 for CMNPD21460, and 52.04 for CMNPD1510, fitting the theoretical range of 0–140. Regarding water solubility (logS), Cpd 4 had a value of −4.01, CMNPD31124 was −5.14, CMNPD21460 was −4.22, and CMNPD1510 was −2.87. While CMNPD31124 approached the lower limit of −5, all values remained within acceptable ranges. Both the octanol/water partition coefficient (logP) and the distribution coefficient (logD) were within the theoretical range of −2 to 5, with higher values observed for Cpd 4 and CMNPD31124 compared to CMNPD21460 and CMNPD1510.

In conclusion, the physicochemical parameters of all compounds fell within theoretical ranges, indicating reasonable structural characteristics suitable for further analysis and research.

### 3.2 Molecular docking-based affinity evaluation and interaction mode analysis

**FIGURE 2:**
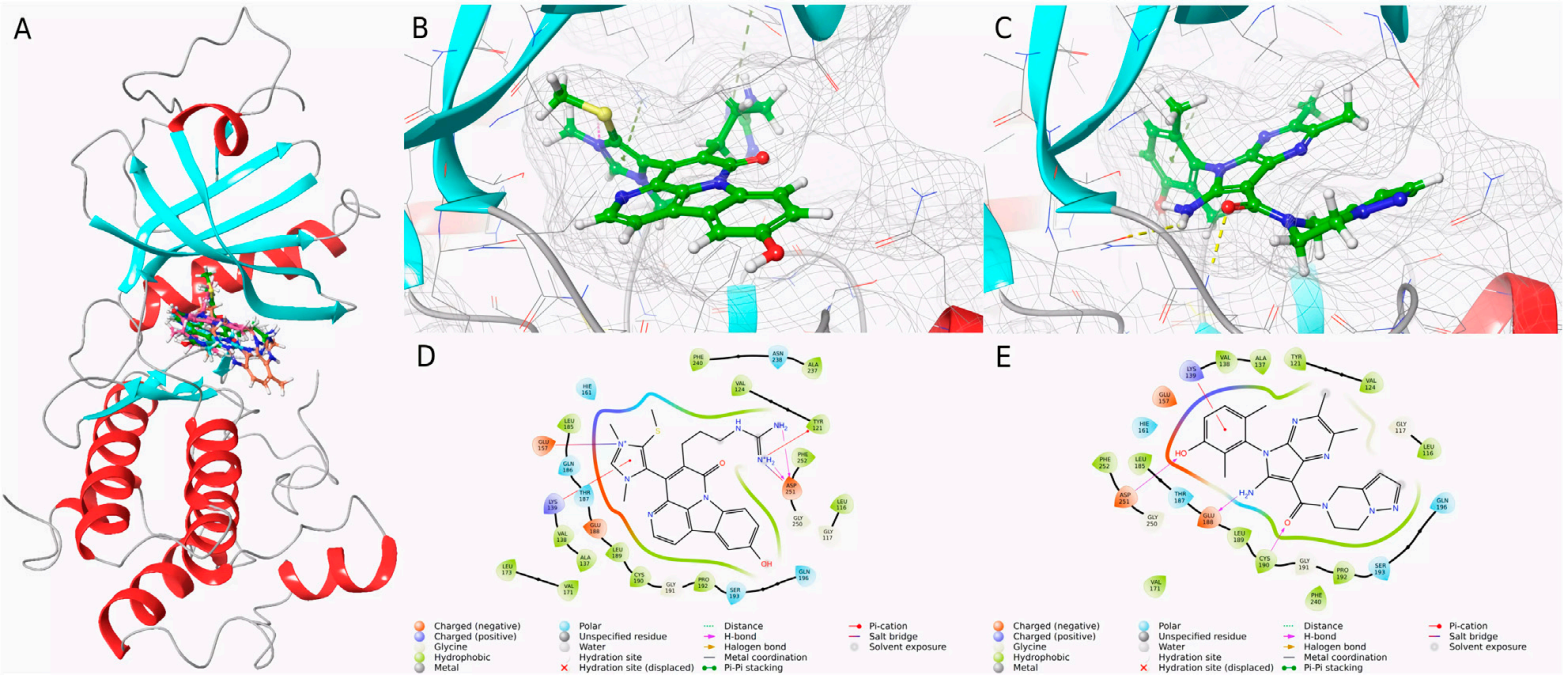
Binding conformations and interaction analysis of selected compounds with PKMYT1. **(A)** Complex conformations of the four compounds bound to the PKMYT1 pocket. **(B)** Close-up view of CMNPD31124 bound to PKMYT1. **(C)** Close-up view of Cpd 4 bound to PKMYT1. **(D)** Interaction diagram of CMNPD31124 with PKMYT1. **(E)** Interaction diagram of Cpd 4 with PKMYT1.

To compare the binding affinities of the three screened compounds with Cpd 4 to PKMYT1, molecular docking analysis was conducted. As shown in Figure 2A, all four compounds shared an identical binding region. According to the docking scores presented in Table 1, CMNPD31124 achieved the lowest score of −9.019, indicating the strongest binding affinity among all compounds. Cpd 4 scored −8.812, slightly higher than CMNPD31124, but still demonstrated considerable binding capability. In contrast, CMNPD1510 and CMNPD21460 scored −6.699 and −6.439, respectively, significantly higher than Cpd 4, reflecting their relatively weaker binding affinities. Consequently, after excluding compounds with weaker binding affinities than Cpd 4, CMNPD31124 emerged as the top candidate, potentially exhibiting superior activity compared to Cpd 4.

**TABLE 1:** Molecular docking scores and binding energies of selected compounds.

Compound ID	Docking score	State penalty	Ligand strain energy	MMGBSA	MMGBSA (NS)
CMNPD31124	−9.019	0.3159	20.475	−71.51	−91.98
Cpd 4	−8.812	0.043	16.977	−59.72	−76.7
CMNPD1510	−6.699	None	None	None	None
CMNPD21460	−6.439	None	None	None	None

Further analysis of docking scores and associated energy parameters revealed that the strain penalty of CMNPD31124 was 0.3159, higher than that of Cpd 4 at 0.043. This suggests that Cpd 4 adopts a binding conformation closer to its native state, potentially offering higher stability. However, regarding ligand strain energy, CMNPD31124 exhibited a value of 20.475, slightly higher than Cpd 4’s 16.977, indicating greater conformational energy required during binding. Nonetheless, in terms of binding free energy, CMNPD31124 demonstrated a significant advantage, with an MMGBSA value of −71.51 kcal/mol, substantially lower than Cpd 4’s −59.72 kcal/mol, indicating stronger binding capability. After nonpolar solvent correction, CMNPD31124s MMGBSA (NS) value was −91.98 kcal/mol, markedly superior to Cpd 4’s −76.7 kcal/mol, further confirming its enhanced binding stability in hydrophobic environments.

Subsequent interaction analyses, illustrated in Figures 2B–E, identified key interaction sites between CMNPD31124 and PKMYT1, including LYS-139, GLU-157, TYR-121, and ASP-251. Among these, LYS-139 and TYR-121 were ATP-binding sites, and ASP-251 served as a magnesium-binding site. In contrast, the interaction sites for Cpd 4 included ASN-238, PHE-240, and LYS-139, with only one ATP-binding site interacting with PKMYT1. These findings indicate that CMNPD31124 not only surpasses Cpd 4 in binding strength but also exhibits a broader interaction range with the target, strongly supporting its potential application at the PKMYT1 target site.

### 3.3 Toxicity prediction and safety assessment of CMNPD31124

Computational toxicity predictions for CMNPD31124, summarized in Supplementary Table S1, suggest that the compound may exhibit multiple potential toxic effects. The predictions indicate a high probability of hepatotoxicity (0.69), neurotoxicity (0.87), respiratory toxicity (0.98), immunotoxicity (0.96), and ecotoxicity (0.73). Additionally, CMNPD31124 is predicted to show inhibitory activity against aromatase (1.00) and activation of estrogen receptor alpha (ER, 0.99) and its ligand-binding domain (ER-LBD, 1.00), suggesting a possible endocrine-disrupting potential. Regarding metabolic interactions, the compound may inhibit acetylcholinesterase (AChE, 0.69), cytochrome CYP2C9 (0.56), and cytochrome CYP3A4 (0.71). These computational predictions suggest that CMNPD31124 may interact with multiple toxicity-related biological targets, particularly endocrine and metabolic pathways, which should be further examined through experimental validation.

Conversely, CMNPD31124 is predicted to have relatively low risks for nephrotoxicity (0.90), cardiotoxicity (0.77), carcinogenicity (0.62), mutagenicity (0.97), and cytotoxicity (0.93). The compound is also predicted to safely cross the blood-brain barrier (BBB, 1.00) and exhibits a low probability of clinical toxicity (0.56) and nutritional toxicity (0.74). Furthermore, the predictions indicate that CMNPD31124 is inactive against several key biological receptors and enzymes, including the aryl hydrocarbon receptor (AhR, 0.97), androgen receptor and its ligand-binding domain (AR and AR-LBD, both 0.99), peroxisome proliferator-activated receptor gamma (PPARγ, 0.99), and mitochondrial membrane potential (MMP, 0.70).

Additionally, the compound is predicted to exhibit minimal interaction risks with p53 (0.96), thyroid hormone receptors α and β (0.90 and 0.78, respectively), glutamate and γ-aminobutyric acid receptors (GABAR and NMDAR, 0.96 and 0.92, respectively), and several cytochrome enzymes (e.g., CYP1A2, CYP2C19, CYP2E1, all with prediction probabilities above 0.76). These predictions suggest that while CMNPD31124 demonstrates computationally favorable safety profiles for certain toxicological targets, further structural optimization may be necessary to mitigate potential toxicity risks, particularly in endocrine and metabolic pathways.

It is important to emphasize that these findings are solely based on computational predictions and do not provide direct experimental validation. Future studies should focus on *in vitro* and *in vivo* assessments to determine the actual biological impact of CMNPD31124 and to validate these toxicity and safety predictions.

### 3.4 Binding stability assessment based on molecular dynamics simulations

**FIGURE 3:**
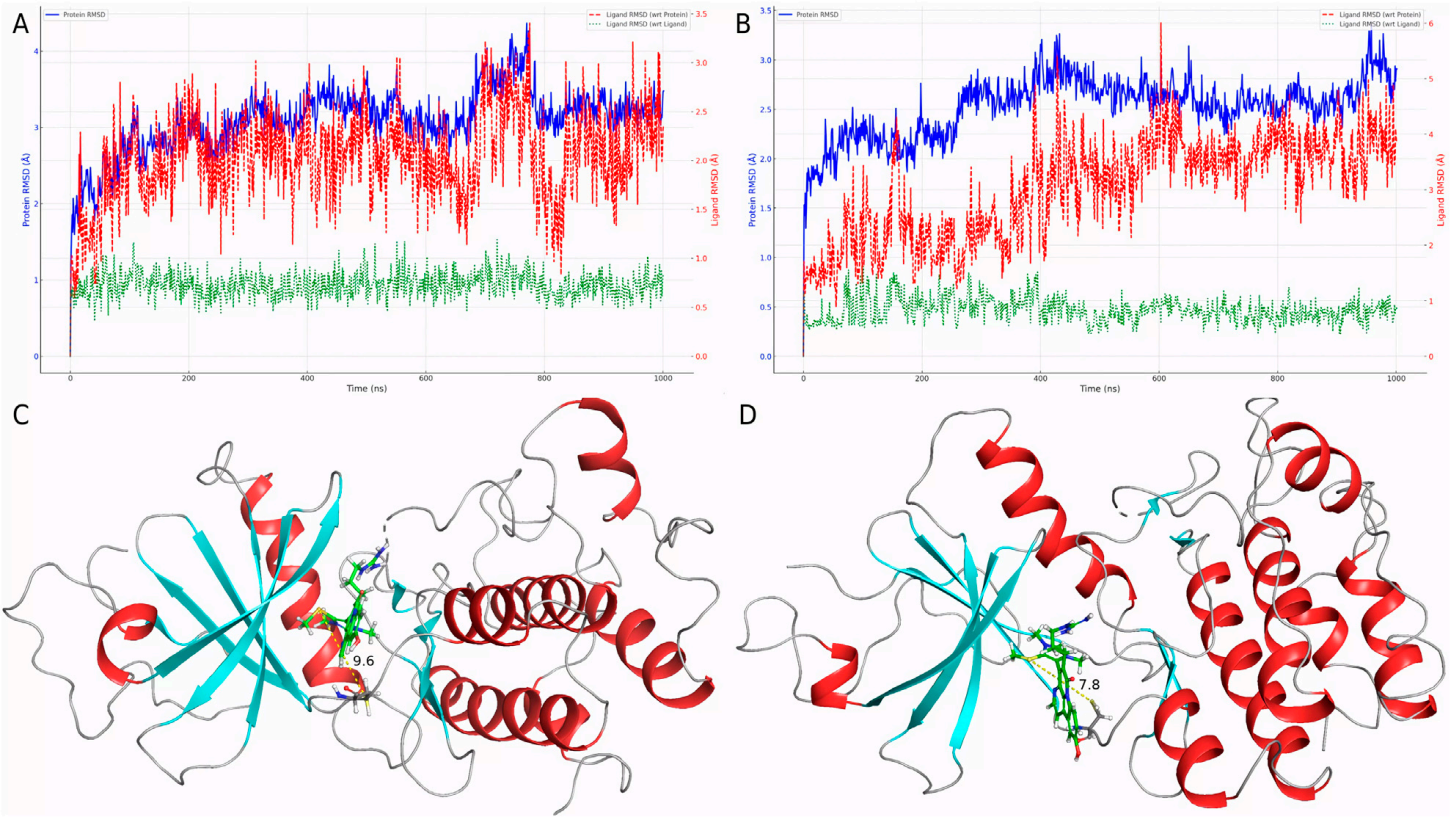
Dynamic behavior and conformational changes of Cpd 4 and CMNPD31124 during MD simulations. **(A)** Time-dependent RMSD profiles of Cpd 4, including ligand RMSD relative to itself, the protein, and the Cα atoms of PKMYT1. **(B)** Time-dependent RMSD profiles of CMNPD31124, including ligand RMSD relative to itself, the protein, and the Cα atoms of PKMYT1. **(C)** Pre-simulation conformation of CMNPD31124 bound to PKMYT1. **(D)** Post-simulation conformation of CMNPD31124 bound to PKMYT1.

To evaluate the dynamic behavior of Cpd 4 and CMNPD31124, MD simulations with identical parameters were conducted for both compounds. The RMSD analysis results (Figures 3A, B) clearly indicate that the binding stability of Cpd 4 is superior to that of CMNPD31124. Throughout the simulation, the ligand RMSD of Cpd 4 relative to itself (Lig_wrt_Ligand) consistently remained at a low level with minimal fluctuations, indicating that its conformation underwent negligible changes during the simulation. Similarly, the ligand RMSD of Cpd 4 relative to the protein (Lig_wrt_Protein) exhibited slight initial fluctuations before rapidly stabilizing, reflecting that the relative position of the binding site remained consistent and the initial binding conformation was close to optimal.

In contrast, CMNPD31124 showed higher initial ligand RMSD relative to itself, which gradually decreased and stabilized over time. This suggests that the initial binding conformation of CMNPD31124 may not have been ideal, requiring significant conformational adjustments during the simulation. Additionally, the RMSD of CMNPD31124 relative to the protein displayed more pronounced initial fluctuations, indicating greater positional changes at the binding site during the early stages of the simulation. This further supports the hypothesis that CMNPD31124 required the simulation process to achieve a more stable conformation.

The RMSD of the protein Cα atoms (Prot_CA) corroborated these findings. When bound to Cpd 4, the protein structure stabilized quickly during the early stages of the simulation. In contrast, the Prot_CA RMSD for CMNPD31124 exhibited more significant fluctuations, potentially reflecting adaptive changes in the protein binding pocket as CMNPD31124 adjusted its binding conformation.

Interestingly, an analysis of the conformational changes before and after the simulation revealed that the -S-CH3 group of CMNPD31124 consistently moved closer to CYS-190 during the simulation. This dynamic behavior suggests that CMNPD31124 may form a covalent bond with CYS-190 *via* disulfide bond formation under physiological conditions. This potential covalent binding capability may compensate for its suboptimal initial binding conformation to some extent, providing valuable clues for further investigation of its mechanism of action.

### 3.5 Modeling of the CMNPD31124-PKMYT1 complex and structure-activity relationship model construction using Chai-1

**FIGURE 4:**
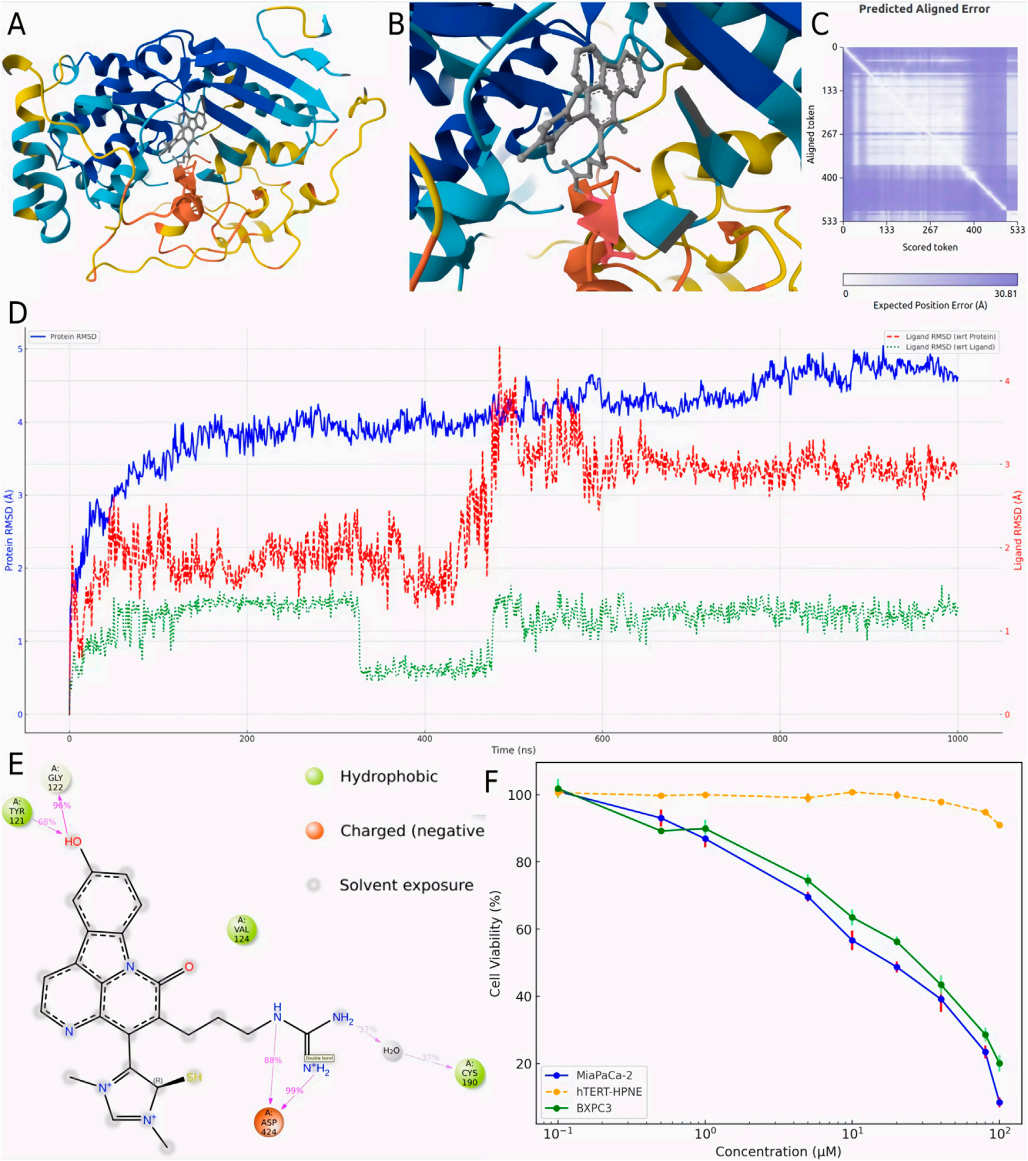
Modeling, stability validation, and interaction analysis of the covalent PKMYT1-CMNPD31124 complex. **(A)** Overall conformation of CMNPD31124 covalently bound to PKMYT1. **(B)** Close-up view of the covalent binding site. **(C)** Predicted Aligned Error (PAE) map for the PKMYT1-CMNPD31124 complex. **(D)** Time-dependent RMSD profiles of the PKMYT1-CMNPD31124 covalent binding model, including ligand RMSD relative to itself, the protein, and the Cα atoms of PKMYT1. **(E)** Interaction diagram illustrating the covalent binding mode of CMNPD31124 with PKMYT1. **(F)** CCK-8 Assay in MiaPaCa-2, BXPC3, and hTERT-HPNE cell lines.

To validate the hypothesis proposed earlier, *de novo* modeling of the CMNPD31124-PKMYT1 complex was performed using Chai-1. The modeling results, presented in Figures 4A, B, confirmed that the -S-CH3 group of CMNPD31124 formed a disulfide bond with CYS-190 of PKMYT1, with a high overall confidence level (Figure 4C). This finding provides structural evidence supporting the covalent interaction between the compound and the protein.

Subsequently, molecular dynamics (Bagheri-Yarmand et al., 2006) simulations were conducted under identical conditions to evaluate the stability of the covalently bonded CMNPD31124 and to construct a structure-activity relationship (SAR) model. The dynamic changes in CMNPD31124s RMSD during the simulation revealed notable trends. The protein Cα RMSD increased rapidly during the initial phase of the simulation and then stabilized, indicating that PKMYT1 underwent swift adaptive adjustments upon binding CMNPD31124, followed by sustained stability. The ligand RMSD relative to the protein exhibited significant initial fluctuations, gradually decreased, and eventually stabilized, suggesting that CMNPD31124 improved its binding stability after positional adjustments at the binding site. Although minor fluctuations persisted in the later simulation stages, the overall trend demonstrated progressive optimization of CMNPD31124s fit within the binding site. Meanwhile, the ligand’s intrinsic RMSD remained consistently low, with minimal variation, indicating that CMNPD31124 maintained a highly stable conformation throughout the simulation.

Further statistical analysis of CMNPD31124s interaction sites during the simulation, illustrated in Figure 4D, revealed the formation of hydrogen bonds with TYR-121 and GLY-122 and a salt bridge with ASP-424. In addition to its direct disulfide bond with CYS-190, CYS-190 also mediated additional interactions with CMNPD31124 through a water bridge. These findings suggest that CMNPD31124 establishes a rich interaction network during binding, reflecting not only robust binding stability but also its potential as a promising candidate compound.

### 3.6 Assessment of cell viability using the CCK-8 assay in MiaPaCa-2, BXPC3, and hTERT-HPNE cell lines

To validate the antitumor activity of CMNPD31124 at the cellular level and confirm that this activity is not due to general cytotoxicity, CCK-8 assays were performed on MiaPaCa-2, BXPC3, and hTERT-HPNE cells. The cells were treated with CMNPD31124 for 48 h, and the results are shown in Figure 4F. MiaPaCa-2 cells exhibited the most pronounced response to the compound, with cell viability decreasing significantly as the drug concentration increased. The IC_50_ for MiaPaCa-2 was approximately 18.6 μM, and further increases in concentration resulted in near-complete loss of viability, reflecting a potent inhibitory effect. In contrast, BXPC3 cells showed a smaller decline in viability, with an IC_50_ of approximately 31.7 μM, suggesting slightly lower sensitivity to CMNPD31124 compared to MiaPaCa-2 cells. At the same concentrations, BXPC3 cells generally maintained higher viability compared to MiaPaCa-2 cells, indicating that BXPC3 cells are less sensitive to the drug. As a normal cell model, hTERT-HPNE cells exhibited minimal change in viability at lower concentrations, demonstrating high tolerance to the compound. Only at concentrations exceeding 80 μM did viability begin to decrease slightly, with the overall reduction remaining mild, indicating low toxicity to normal cells. Overall, both MiaPaCa-2 and BXPC3 cells showed clear dose-dependent inhibition following 48 h of treatment, while hTERT-HPNE cells remained largely unaffected at lower doses, clearly illustrating the differential cellular responses to CMNPD31124.

### 3.7 RMSD analysis of four mutants (Y121A, G122A, C190A, and D424A) and their impact on binding stability

**FIGURE 5:**
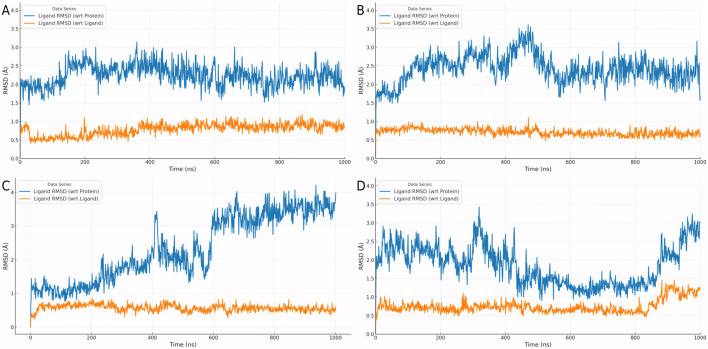
Dynamic RMSD profiles of ligand binding in four PKMYT1 mutants. **(A)** Y121A **(B)** G122A **(C)** C190A **(D)** D424A.

The RMSD analysis of the four mutants—Y121A, G122A, C190A, and D424A—provides further insights into the differences in binding stability, as illustrated in Figures 5A–D. In the Y121A mutant (Figure 5A), the ligand’s RMSD exhibited noticeable fluctuations during the initial stages but gradually decreased and stabilized. This indicates that the Y121A mutation caused some disruption to the binding mode but had minimal impact on the overall conformational stability of the ligand. For the G122A mutant (Figure 5B), the ligand RMSD showed only minor fluctuations early in the simulation and quickly stabilized, suggesting that the G122A mutation had a weaker impact on the binding environment, allowing the ligand to adapt rapidly and maintain a stable binding state.

In the case of the C190A mutant (Figure 5C), the ligand RMSD initially exhibited significant fluctuations but eventually decreased to a relatively stable range, albeit with ongoing dynamic changes. This indicates that the C190A mutation substantially altered the binding environment, requiring the ligand to undergo continuous conformational adjustments to adapt to the new binding mode. In contrast, the D424A mutant (Figure 5D) showed large initial RMSD fluctuations, and although these decreased to a smaller range, complete stabilization was not achieved. This reflects that the D424A mutation caused the most pronounced disruption to the binding environment, with the ligand undergoing persistent dynamic adjustments throughout the simulation.

Overall, Figures 5A–D demonstrate significant differences in the effects of various mutations on ligand binding stability and adaptability. Mutations Y121A and G122A exerted minor impacts, allowing the ligand to adapt rapidly and maintain stable binding. In contrast, mutations C190A and D424A caused greater perturbations in the binding environment, requiring the ligand to undergo more extensive conformational adjustments. These findings provide important insights into the structure-activity relationship and serve as a valuable foundation for optimizing the binding mode.

### 3.8 Stability assessment of covalent CMNPD31124-PKMYT1 complex in intracellular and in vivo environments

**FIGURE 6:**
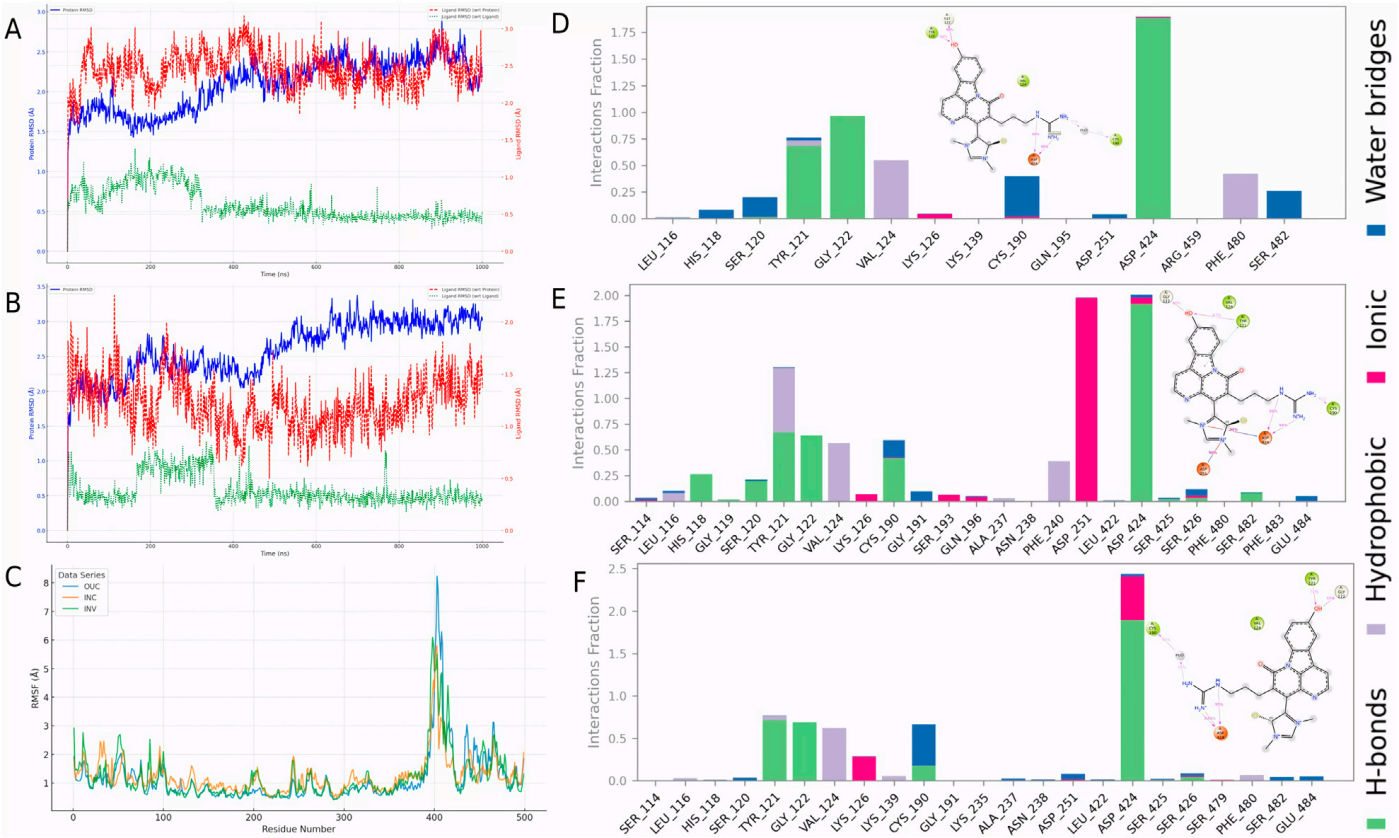
Dynamic stability and interaction analysis of the covalent PKMYT1-CMNPD31124 complex across different simulation environments. **(A)** Time-dependent RMSD profiles of the PKMYT1-CMNPD31124 covalent binding model in the intracellular simulation environment. **(B)** Time-dependent RMSD profiles of the PKMYT1-CMNPD31124 covalent binding model in the *in vivo* simulation environment. **(C)** RMSF analysis of PKMYT1 in extracellular, intracellular, and *in vivo* simulation environments. **(D–F)** Interaction frequency of PKMYT1-CMNPD31124 in **(D)** extracellular, **(E)** intracellular, and **(F)** *in vivo* environments.

To evaluate the stability of CMNPD31124 after covalent binding to PKMYT1, simulations were conducted in intracellular and *in vivo* environments. In the intracellular environment (Figure 6A), the protein Cα RMSD exhibited a slight initial increase before stabilizing, indicating that the overall structure of PKMYT1 gradually achieved stability. The ligand RMSD relative to the protein demonstrated dynamic adjustments throughout the simulation, while the ligand’s intrinsic RMSD showed minimal variation, reflecting the good conformational stability and binding adaptability of CMNPD31124 in the intracellular environment. In the *in vivo* environment (Figure 6B), the protein Cα RMSD stabilized rapidly, and the ligand RMSD relative to the protein, after initial fluctuations, also reached stability. The ligand’s intrinsic RMSD consistently remained low, further confirming its high binding stability and adaptability in the *in vivo* setting.

Additionally, RMSF analysis (Figure 6C) revealed significant differences in protein residue flexibility after CMNPD31124 covalently bound to PKMYT1 across extracellular (Stading et al., 2020), intracellular (Su et al., 2020), and *in vivo* (INV) environments. RMSF values were highest in the extracellular environment, with pronounced fluctuations for some residues, likely attributable to model optimization during the simulation, which enhanced residue flexibility and localized structural adjustments. In contrast, RMSF values in the intracellular and *in vivo* environments were lower, indicating greater overall protein stability in these contexts. The intracellular environment showed the lowest RMSF values, suggesting that PKMYT1 experienced more restricted dynamic changes upon binding CMNPD31124, reflecting higher structural stability. RMSF values in the *in vivo* environment were intermediate, with moderate residue flexibility observed, indicating that the protein retained dynamic adaptability in the complex *in vivo* environment while maintaining stability.

Further analysis of CMNPD31124s interacting residues across the three environments (Figures 6E, F) identified high-frequency interaction sites, including TYR-121, GLY-122, VAL-124, CYS-190, and ASP-424. Notably, in the intracellular environment, ASP-251 exhibited a significantly increased interaction frequency, suggesting that the intracellular setting might induce unique molecular interactions. These findings demonstrate that CMNPD31124 can maintain critical interactions across diverse environments, while environmental differences may also facilitate novel interaction patterns, enhancing its binding stability and adaptability. These results support the further optimization of CMNPD31124 and underscore its potential as a therapeutic candidate compound.

### 3.9 Analysis of CMNPD31124s dynamic behavior across different simulation environments

**FIGURE 7:**
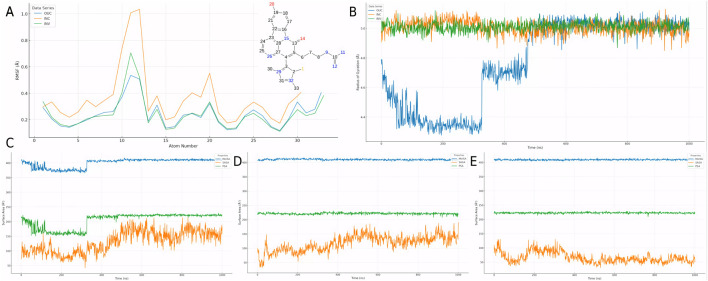
Dynamic behavior of CMNPD31124 across simulation environments. **(A)** RMSF of CMNPD31124 in extracellular, intracellular, and *in vivo* environments. **(B)** Time-dependent Rg of CMNPD31124 across the three environments. **(C–E)** Time-dependent area changes of CMNPD31124 in **(C)** extracellular, **(D)** intracellular, and **(E)** *in vivo* environments, including solvent-accessible surface area (SASA), molecular surface area (MolSA), and polar surface area (PSA).

To analyze the dynamic behavior of CMNPD31124 in various simulation environments, RMSF, radius of gyration (Rg), and surface area metrics were evaluated for the extracellular (Stading et al., 2020), intracellular (Su et al., 2020), and *in vivo* (INV) environments. The RMSF analysis of CMNPD31124 (Figure 7A) revealed clear differences in ligand flexibility across environments. In the extracellular environment, RMSF values were relatively low, indicating high conformational stability and restricted flexibility. In the intracellular environment, RMSF values increased, with significant fluctuations observed in specific regions, reflecting enhanced ligand flexibility. In the *in vivo* environment, RMSF values were intermediate, suggesting that the ligand achieved a dynamic balance in flexibility. The formation of a salt bridge with ASP-251 in the intracellular environment may have resulted from increased ligand flexibility, allowing specific groups to be exposed and form interactions. This highlights the ligand’s adaptability and binding advantages in dynamic conditions.

The Rg analysis across the three environments (Figure 7B) further illustrated changes in protein compactness. In the extracellular environment, Rg values were low with minimal fluctuations, indicating that the protein maintained high compactness after initial model optimization. In the intracellular environment, Rg values were slightly higher with increased fluctuations, suggesting more complex intermolecular interactions and enhanced local structural flexibility. In the *in vivo* environment, Rg values were the highest, reflecting a balance between protein flexibility and compactness, which supports adaptation to the complex biological milieu.

Surface area metrics, including solvent-accessible surface area (SASA), molecular surface area (MolSA), and polar surface area (PSA), were also analyzed across environments (Figures 7C–E). In the extracellular environment, SASA increased significantly due to the effects of model optimization, while MolSA and PSA remained stable, indicating that the compound underwent dynamic adjustments to adapt to initial conditions. In the intracellular environment, SASA decreased, with MolSA and PSA further stabilizing, suggesting tighter binding and reduced flexibility. In the *in vivo* environment, SASA was intermediate, with minor fluctuations in MolSA and PSA, indicating that the compound retained necessary flexibility while maintaining high structural stability in the complex environment.

In summary, CMNPD31124 demonstrated excellent adaptability and binding stability across the three environments. RMSF, Rg, and surface area metrics collectively support the ligand’s potential to maintain binding efficiency and structural integrity in dynamic conditions. These findings provide a theoretical foundation for CMNPD31124s development as a promising therapeutic candidate.

## 4 Discussion

In the treatment of pancreatic ductal adenocarcinoma (PDAC), although targets such as KRAS and BRCA mutations have received considerable attention, their clinical application is often constrained by mutation frequencies or limitations to specific patient subgroups (Wei and Ren, 2024; Sarantis et al., 2020). In contrast, PKMYT1, a non-mutant target, is highly overexpressed in PDAC and closely associated with poor prognosis (Wang et al., 2024a). Its inhibition significantly suppresses tumor cell proliferation and induces apoptosis. Beyond its classical role in cell cycle regulation, PKMYT1 uniquely participates in tumorigenesis by modulating PLK1, showcasing a broader therapeutic potential compared to traditional targets (Liu et al., 2020). Studies have demonstrated that PKMYT1 inhibitors, such as RP-6306, exhibit potent antitumor activity in PDAC cell lines and patient-derived xenograft models with minimal toxicity (Szychowski et al., 2022). These findings underscore the feasibility and versatility of PKMYT1 as a drug development target, offering advantages over KRAS and similar targets.

CMNPD31124 (also referred to as Compound 1,211 or Ishigadine A) is a novel indole alkaloid derived from marine sponges of genera such as Acanthostrongylophora, Dragmacidon, Hyrtios, and Oceanapia (Takahashi et al., 2018). It features a complex cyclic skeleton and distinctive functional group modifications, marking it as a natural product with significant pharmacological potential. Notably, CMNPD31124 has been reported to exhibit moderate cytotoxicity against L1210 murine leukemia cells, with an IC_50_ value of 3.3 μg/mL, while demonstrating no significant cytotoxicity against KB human epidermoid carcinoma cells (IC_50_ > 10 μg/mL). This selective cytotoxicity underscores its potential in targeted cancer therapy. While its specific bioactivities remain largely unexplored, CMNPD31124 shares properties commonly associated with indole alkaloids, such as anticancer, antibacterial, antiviral, and anti-inflammatory activities (Omar et al., 2021). Its discovery highlights the diversity and uniqueness of marine natural products as sources of drug discovery and suggests potential for rational design targeting cancer-associated proteins like PKMYT1. Moreover, the CCK-8 assay further validated the antitumor activity of CMNPD31124 in PDAC cell lines (Section 3.6). The results demonstrated that CMNPD31124 exhibited an IC_50_ of 18.6 μM in MiaPaCa-2 cells and 31.7 μM in BXPC3 cells, while normal pancreatic hTERT-HPNE cells remained largely unaffected at concentrations up to 80 μM. This selective inhibition indicates that CMNPD31124 has a strong cytotoxic effect on PDAC cells while exerting minimal toxicity on normal pancreatic cells, further supporting its potential as a promising therapeutic candidate.

Molecular dynamics simulations in this study revealed that CMNPD31124 undergoes significant conformational rearrangements during the binding process and ultimately establishes a stable binding network with PKMYT1. These dynamic adjustments reflect the inherent flexibility of the protein binding site and the adaptability of the ligand (Antunes et al., 2015). While the initial docking-derived conformation represents a theoretical best-fit under static conditions, the final binding conformation observed in MD simulations highlights the critical role of protein-ligand dynamics in drug discovery (Salmaso and Moro, 2018). Further analysis showed that CMNPD31124 forms strong hydrogen bonds and hydrophobic interactions with key PKMYT1 residues, including CYS-190 and TYR-121, enhancing its binding stability and demonstrating its potential as a PKMYT1-targeted inhibitor.

Chai-1 demonstrates advantages over AlphaFold3 (AF3) (Abramson et al., 2024) and RoseTTAFold All-Atom (RFAA) (Krishna et al., 2024) in capturing detailed interactions at small-molecule binding sites. It provides accurate modeling of hydrogen bonds, hydrophobic interactions, and π-π stacking, while also predicting covalent inhibitory conformations. For example, Chai-1 successfully identified a covalent bond between CMNPD31124 and the CYS-190 residue of PKMYT1, supported by additional interactions with TYR-121 and GLY-122. These features highlight its utility in structural analysis and covalent inhibitor design.

However, a limitation of Chai-1 is that it can sometimes correctly predict the individual chains in a complex, but fail to place them in the correct relative orientations. This issue is particularly relevant when dealing with complex protein-ligand interactions, as seen in Figure 4, where the predicted complex lacked the proper structural context without additional contact information. Another limitation is Chai-1’s sensitivity to modified residues. When modified residues are removed or replaced with their unmodified analogs, the predicted structures can undergo significant changes. This suggests that Chai-1 relies on modification-specific information to generate accurate models, and predictions may vary when these modifications are absent. Despite these limitations, Chai-1 remains a valuable tool for predicting covalent binding interactions and offers valuable insights for the rational design of PKMYT1 inhibitors. Additionally, as a web-based platform with low system requirements, Chai-1 ensures accessibility and ease of use, making it a practical tool for early-stage drug discovery. Covalent inhibitors have demonstrated significant clinical value in cancer therapy (Zheng et al., 2023). For example, the third-generation EGFR inhibitor Osimertinib (La Monica et al., 2019) and the KRAS G12C inhibitor AMG 510 (Canon et al., 2019) have achieved remarkable success in overcoming drug resistance and enhancing target specificity. Similarly, CMNPD31124s ability to form a covalent bond with PKMYT1 highlights its potential to address therapeutic challenges associated with traditional targets.

While CMNPD31124 demonstrates promising therapeutic potential, its toxicity profile requires careful consideration. Toxicity predictions indicate potential risks for hepatotoxicity, neurotoxicity, respiratory toxicity, immunotoxicity, and endocrine disruption due to its high activity against aromatase and estrogen receptors. Notably, neurotoxicity may involve microglia-mediated neuroinflammation, which has been linked to cardiovascular diseases through alterations in autonomic nervous system activity (Wang et al., 2022a). These findings suggest that further *in vitro* and *in vivo* studies are needed to clarify the impact of CMNPD31124 on neuroimmune interactions, particularly in the context of neuroinflammation and cardiovascular risks. These effects may result from nonspecific binding to critical proteins, disrupting metabolic enzymes, immune responses, and hormonal signaling (Johnson and Hummer, 2011; Hu et al., 2024; Xu et al., 2024b). Additionally, its inhibition of CYP2C9, CYP3A4, and acetylcholinesterase raises concerns about impaired drug metabolism and neurotoxicity. The potential for these toxicities emphasizes the need for careful structural optimization of CMNPD31124 (Kumar et al., 2006; Chen et al., 2023).

To mitigate these risks, we propose a strategy for structural optimization to reduce nonspecific binding to CYP enzymes and estrogen receptors, which are implicated in metabolic and endocrine disruptions. Molecular docking and dynamics simulations can be used to design derivatives with higher specificity and reduced off-target binding. These computational methods can guide the rational design of safer analogs by targeting key binding sites that are less likely to disrupt vital metabolic pathways.

While the toxicity predictions provide useful guidance, *in vitro* toxicity screening using relevant cell lines, such as HepG2 cells (liver model) and PC12 cells (neurotoxicity model), will provide more accurate insights into the compound’s safety profile. These assays can evaluate the compound’s effects on cell viability, metabolic enzymes, and signaling pathways. Additionally, early-stage pharmacokinetic studies, including metabolic stability and CYP enzyme inhibition, would be useful to refine the compound’s drug-like properties and identify any significant metabolic liabilities before moving into *in vivo* testing. Despite these promising findings, the lack of experimental validation remains a limitation of this study. While computational techniques such as virtual screening, molecular docking, and MD simulations provide valuable theoretical insights, further *in vitro* and *in vivo* studies are essential to evaluate CMNPD31124s efficacy, pharmacokinetics, and toxicity.

It is also important to acknowledge the limitations of the computational methods used in this study. While molecular docking and molecular dynamics simulations offer valuable insights into the binding affinity and dynamic behavior of CMNPD31124, the accuracy of these methods is influenced by several factors. For example, docking scores may not always correlate perfectly with experimental binding constants due to the flexibility of the ligand and receptor, and the force field used in molecular dynamics simulations may not fully capture all interactions in complex biological environments. Furthermore, while the Chai-1 modeling framework provides accurate predictions of covalent binding, its predictions are dependent on the quality of the input structures and the assumptions made during modeling. These limitations highlight the importance of experimental validation in confirming the predictions made by computational methods.

In conclusion, this study integrates advanced modeling and simulation approaches to uncover the dynamic binding mechanisms of CMNPD31124 with PKMYT1. The findings not only validate the compound’s therapeutic potential but also establish a foundation for the design and development of optimized inhibitors targeting PKMYT1.

## 5 Conclusion

This study identified CMNPD31124 as a promising PKMYT1 inhibitor with strong binding affinity and covalent interaction potential. *In vitro* assays demonstrated selective cytotoxicity, with IC_50_ values of 18.6 μM in MiaPaCa-2 and 31.7 μM in BXPC3 cells, while normal pancreatic hTERT-HPNE cells remained largely unaffected at concentrations up to 80 μM. These results highlight the potential of CMNPD31124 as a therapeutic candidate for targeting PKMYT1 in cancer cells. However, despite its promising therapeutic potential, predicted toxicity risks, including hepatotoxicity and neurotoxicity, necessitate further structural optimization. Future research should focus on refining the compound’s pharmacological properties through molecular docking and dynamics simulations to reduce off-target effects. In addition, *in vivo* studies in preclinical models are essential to validate the compound’s therapeutic efficacy, pharmacokinetics, and long-term safety. Mechanistic studies, including the evaluation of G2/M checkpoint disruption and chromosomal instability, will provide further insights into the underlying antitumor effects. Moreover, combination therapy approaches could be explored to enhance the efficacy of CMNPD31124 in overcoming resistance and improving treatment outcomes. Ultimately, if preclinical validation supports the findings, clinical trials will be necessary to evaluate its safety and efficacy in humans.

## Data Availability

The raw data supporting the conclusions of this article will be made available by the authors, without undue reservation.
